# Two Cases of Arrhythmic Mitral Valve Prolapse: Evil Has Many Faces

**DOI:** 10.19102/icrm.2022.130505

**Published:** 2022-05-15

**Authors:** Dimitrios Sfairopoulos, Lampros Lakkas, Aidonis Rammos, Panagiotis Korantzopoulos

**Affiliations:** ^1^First Department of Cardiology, Faculty of Medicine, University of Ioannina, Ioannina, Greece; ^2^Second Department of Cardiology, Faculty of Medicine, University of Ioannina, Ioannina, Greece

**Keywords:** Implantable cardioverter-defibrillator, mitral valve prolapse, sudden death, ventricular arrhythmias

## Abstract

Mitral valve prolapse (MVP) is one of the most common valvular heart diseases. Although MVP is generally considered benign, it can be associated with important complications, including sudden cardiac death (SCD), owing to ventricular arrhythmias (VAs). Several clinical, electrocardiographic, and imaging findings have been associated with MVP-related SCD, including female sex, T-wave inversions in the inferior leads, complex ventricular ectopy, leaflet redundancy (classic MVP), mitral annular disjunction, pickelhaube sign (a spiked configuration of the lateral annular velocities), and evidence of myocardial fibrosis in cardiac magnetic resonance (CMR) imaging. However, neither of these markers, nor any specific combination of them, have proved to be a consistent predictor of malignant VAs and SCD. In this context, we present 2 interesting cases of arrhythmic MVP, highlighting the broad clinical spectrum of this condition, the potential underlying arrhythmogenic mechanisms, and the merit of identifying patients at high arrhythmic risk.

## Introduction

Although mitral valve prolapse (MVP) is generally considered a benign valvular heart disease, it can be associated with significant adverse sequelae, including sudden cardiac death (SCD), owing to ventricular arrhythmias (VAs).^1^ Several clinical, electrocardiographic, and imaging findings have been associated with SCD in patients with MVP. However, identifying the small subset of patients who may be at increased risk for a fatal arrhythmic event remains difficult.^2^ In this article, we report 2 interesting cases of arrhythmic MVP, highlighting the broad clinical spectrum of this condition, the potential underlying arrhythmogenic mechanisms, and the merit of identifying patients at high arrhythmic risk.

## Case reports

### Case 1

A 45-year-old female patient was transferred to our hospital after suffering a witnessed cardiac arrest at home. The patient had been in her usual state of good health until 45 minutes before admission, when she collapsed suddenly. Her husband called emergency medical services (EMS) and initiated cardiopulmonary resuscitation (CPR). The EMS personnel identified the presenting heart rhythm as ventricular fibrillation; a 200-J asynchronous shock was delivered, and spontaneous circulation returned. Subsequently, emergency intubation of the trachea was performed.

**Figure 1:**
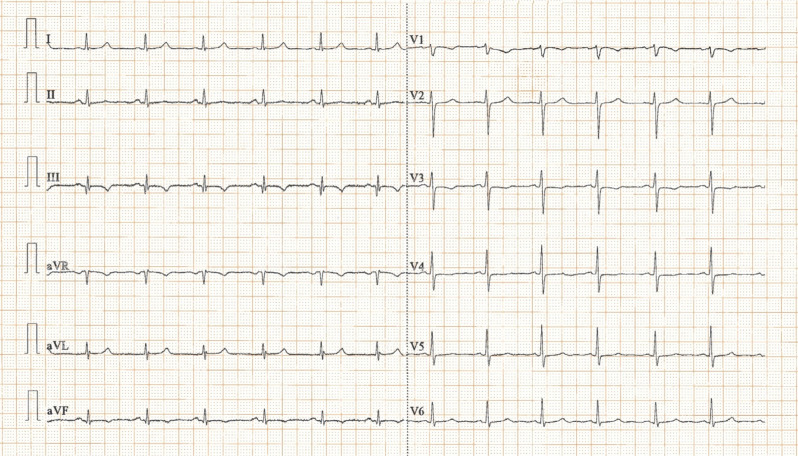
Case 1 electrocardiogram at the time of admission. Note the presence of small Q-waves and T-wave inversions in leads III and aVF.

**Figure 2:**
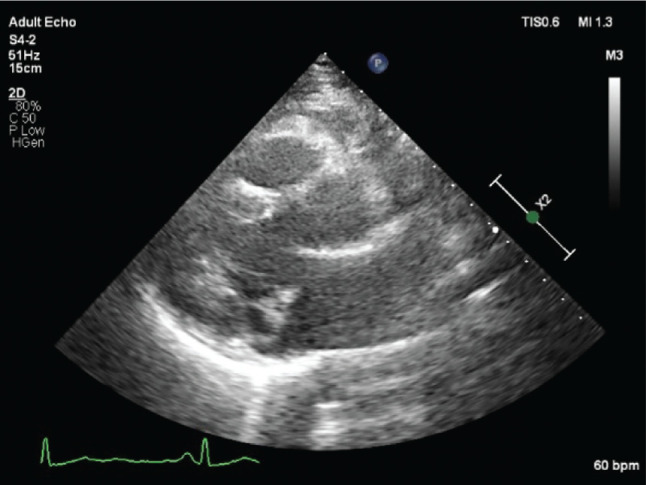
Case 1 echocardiogram showing bileaflet mitral valve prolapse in the context of Barlow’s disease.

Upon arrival to the cardiac intensive care unit of our hospital, the patient was hemodynamically stable and was receiving mechanical ventilatory support. Cardiac auscultation revealed a crisp mid-systolic click best heard over the left apex, but no murmur was noted. The rest of the examination was normal. Her past medical history was unremarkable, and her family history was negative for SCD events. She was not taking any medications, and her husband denied illicit drug or dietary supplement use. An electrocardiogram (ECG) on admission showed sinus rhythm, as well as small Q-waves and T-wave inversions in leads III and aVF **(Figure 1)**. Telemetric monitoring showed frequent polymorphic premature ventricular contractions (PVCs). A complete blood count and biochemical parameters, including electrolytes as well as renal and thyroid function tests, were all within normal limits; a d-dimer level was undetectable, and high-sensitivity troponin I was mildly elevated (<5 times the upper limit of normal). The patient was started on metoprolol. A coronary angiogram showed no evidence of obstructive coronary artery disease. Transthoracic echocardiography revealed bileaflet MVP with redundant leaflets (maximal leaflet thickness of ≥5 mm during diastasis—classic MVP) in the context of Barlow’s disease and mitral annular disjunction (MAD—ventriculo-annular detachment) **(Figure 2)**, but no evidence of mitral regurgitation (MR), flail, rupture chordae, or pickelhaube sign (a spiked configuration of the lateral annular velocities). No further echocardiographic abnormalities were evident.

On the second day of hospitalization, the patient was weaned from ventilation support and, 5 days later, contrast-enhanced cardiac magnetic resonance (CMR) imaging was performed and showed a left ventricle of normal size and function with no regional wall motion abnormalities and no evidence of myocardial focal fibrosis (lack of late gadolinium enhancement). In addition, there was no evidence of hyperemia (lack of early gadolinium enhancement) or edema. Bileaflet MVP, leaflet redundancy, and MAD were confirmed. The right ventricle was of normal size and function, and there was no pericardial effusion.

The aforementioned findings excluded ischemic heart disease as the cause of the cardiac arrest. Furthermore, there was no evidence of cardiomyopathy, and the Lake Louise criteria for the diagnosis of acute myocarditis were not fulfilled. Therefore, it was concluded that arrhythmic MVP was the cause of the cardiac arrest. On the ninth day of hospitalization, an implantable cardioverter-defibrillator was placed for secondary prevention of SCD, and the patient was discharged the day after. She has remained asymptomatic without any arrhythmic events for 20 months after the implantation.

### Case 2

A 59-year-old male patient was referred to our hospital because of severe MR due to bileaflet MVP with flail posterior leaflet. The patient had been in his usual state of good health until 1 month before admission, when he developed palpitations. Two days before admission, he was evaluated at a local hospital. He reported no chest pain, dyspnea, orthopnea, or limitation of his physical activity. His past medical history was unremarkable, and his family history was negative for SCD events. He was not taking any medications and denied illicit drug use or intake of dietary supplements. Physical examination was remarkable for a holosystolic murmur best audible at the apex. There was no evidence of a mid-systolic click. An ECG showed sinus rhythm, without evidence of repolarization abnormalities. Transthoracic echocardiography revealed severe, eccentric MR due to bileaflet MVP (Barlow’s disease) with flail posterior leaflet. Subsequently, the patient was referred to our institution for further evaluation.

**Figure 3:**
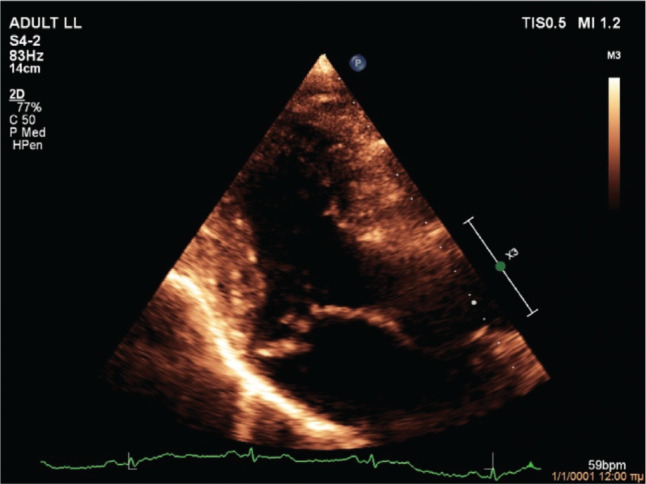
Case 2 echocardiogram showing bileaflet mitral valve prolapse (Barlow’s disease) with flail posterior leaflet.

**Figure 4:**
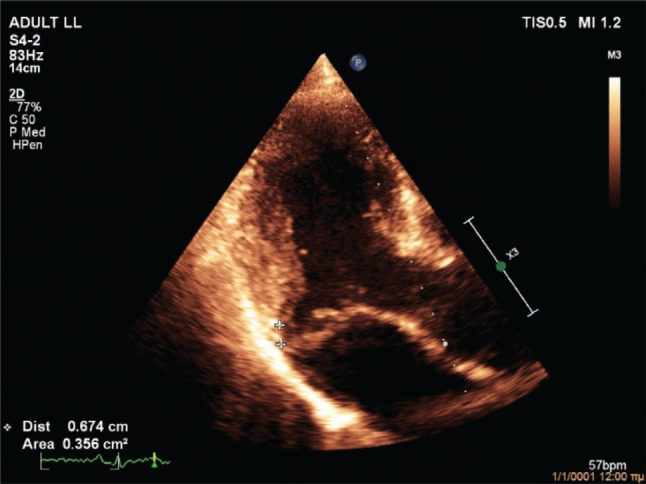
Case 2 echocardiogram showing mitral annular disjunction.

**Figure 5:**
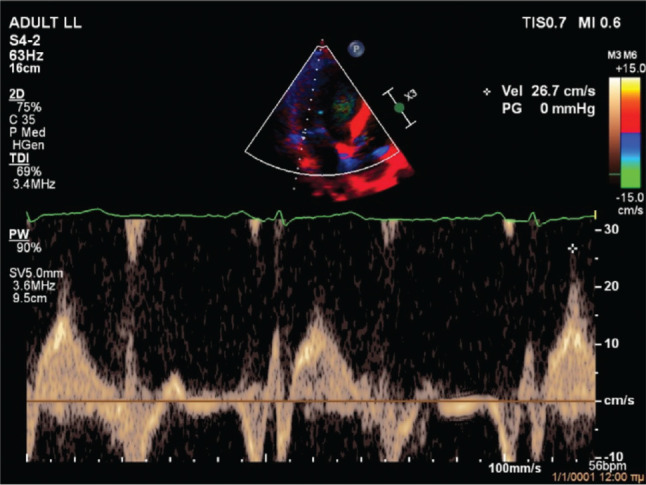
Case 2 tissue Doppler imaging showing pickelhaube sign (peak systolic lateral mitral annulus velocity of ≥16 cm/s resembling the “pickelhaube,” a spiked helmet).

**Figure 6:**
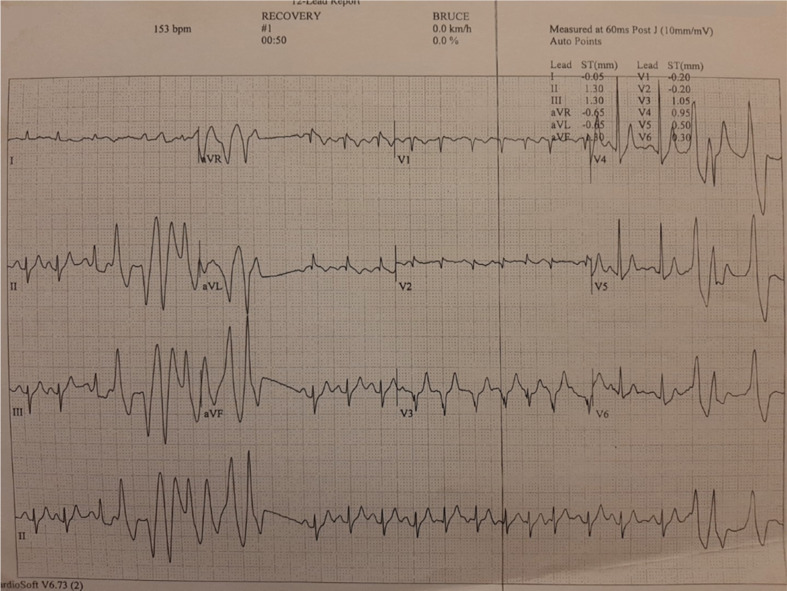
Case 2 complex ventricular ectopy during the treadmill exercise stress test (polymorphic premature ventricular complexes and polymorphic non-sustained ventricular tachycardia).

On evaluation at our hospital, transthoracic echocardiography confirmed the diagnosis of severe, eccentric MR due to bileaflet MVP (Barlow’s disease) with flail posterior leaflet **(Figure 3)**. The left ventricle had an increased end-diastolic diameter of 62 mm, but the end-systolic diameter was normal. The ejection fraction of the left ventricle was 65%. There was no evidence of regional wall motion abnormalities. Importantly, MAD **(Figure 4)** and pickelhaube sign **(Figure 5)** were documented. The remainder of the ECG was unremarkable. A treadmill exercise stress test was performed, during which the patient developed polymorphic PVCs and runs of polymorphic non-sustained ventricular tachycardia (VT) (NSVT) **(Figure 6)**. A complete blood count and biochemical parameters, including electrolytes and tests of renal and thyroid function, were all normal. The patient was started on metoprolol. A coronary angiogram showed no evidence of obstructive coronary artery disease. The patient was referred for CMR imaging and emergent mitral valve repair surgery; unfortunately, he postponed the scheduled procedures and suffered a fatal out-of-hospital cardiac arrest.

## Discussion

MVP encompasses a spectrum of clinical entities characterized by a displacement of one or both mitral leaflets of >2 mm above the mitral annulus during systole.^3^ Based on the thickness of mitral valve leaflets during diastasis, MVP can be classified as classic or non-classic. In classic MVP, mitral leaflets have a maximal thickness of ≥5 mm (redundant leaflets), whereas, in non-classic MVP, the mitral leaflet thickness is <5 mm. From a pathologic perspective, MVP can be subdivided into Barlow’s disease and fibroelastic deficiency. Barlow’s disease results from myxoid infiltration of the mitral leaflets, which become thickened and distended, often leading to multi-segmental prolapse. Conversely, fibroelastic deficiency is caused by impaired production of connective tissue, resulting in leaflet and chordae thinning.^4^

MVP is a common valvular disease in the general population. Using revised echocardiographic diagnostic criteria, the Framingham Heart Study reported prevalence rates of 1.3% for classic and 1.1% for non-classic MVP, respectively, yielding an estimated overall prevalence of 2.4%.^5^ The complications of MVP include severe MR, bacterial endocarditis, heart failure, cardiac arrhythmias, and occasionally SCD.^2^ Although the incidence of MVP-related SCD was initially believed to be trivial, more recent observational data indicate that it may occur more frequently, with an estimated risk of ≥2 times that of the general population, varying from 0.2%–1.9% per year.^6–10^ Relevant risk markers for MVP-related SCD include female sex, T-wave inversions in the inferior leads, complex ventricular ectopy, leaflet redundancy (classic MVP), MAD, pickelhaube sign, and evidence of myocardial fibrosis on CMR imaging.^2^ However, neither of these risk markers on their own, nor any specific combination of them, have proved to be a consistent predictor of malignant VAs and SCD in patients with MVP.^2^

MVP-related malignant VAs are thought to be caused by a complex interplay between a substrate (regional myocardial fibrosis, hypertrophy, Purkinje system dysfunction), a trigger (complex ventricular ectopy), and transient modulators (autonomic system dysfunction, electrolyte abnormalities, altered hemodynamics).^1^

Focal (replacement) fibrosis in the papillary muscles and inferolateral portions of the left ventricle, as can be detected by late gadolinium enhancement on CMR, is highly prevalent among patients with MVP-related SCD and complex VAs.^8^ MVP has also been associated with diffuse myocardial fibrosis, as suggested by reduced postcontrast T1 times, leading to subclinical systolic dysfunction.^11^ Fibrosis may enable triggered activity or re-entry, and thus initiate and/or perpetuate VAs.

Ventricular ectopy, particularly of fascicular or papillary origin, but also that from the outflow tract or the mitral annulus, has been described to serve as a trigger for malignant VAs in patients with arrhythmic MVP.^1^ In general, VAs originating from the posteromedial papillary muscle, posterior fascicle, or the posterior mitral annulus have a superior axis, whereas those originating from the anterolateral papillary muscle, anterior fascicle, or the anterior part of the mitral annulus have an inferior axis.^12^ The QRS interval is wider in VAs originating from the papillary muscles compared to those arising from the fascicles. Fascicular VAs exhibit an rsR’ pattern in lead V1, which is not present in papillary muscle VAs.^12^ Of note, the aforementioned electrocardiographic criteria have potential limitations owing to the fact that papillary muscles have connections with areas located away from their base and thus may produce PVCs with an inconsistent electrocardiographic origin.^12^

According to preclinical models of stretch-induced arrhythmias, mechanical traction and myocardial stretch caused by the prolapsing leaflet may result in local premature ventricular activation,^13^ shortening of the action potential duration, a decrease in resting diastolic potential, and a development of stretch-induced early afterdepolarizations.^14,15^ In addition, prolongation of the ventricular functional refractory period in the traction zone may cause action potential heterogeneities, thereby providing a substrate for polymorphic VT or a trigger for short-coupled PVCs based on the “R-from-T” mechanism.^12,16^ Even further, according to 2-dimensional and 3-dimensional simulations, large heterogeneities have been suggested to induce polymorphic VT due to multiple competing foci and small heterogeneities to result in a re-entry type of polymorphic VT.^17^

As far as the Purkinje system is concerned, it has been suggested that it may serve as both a substrate and a trigger for malignant VAs in patients with arrhythmic MVP. As was shown by a series of consecutive patients with bileaflet MVP and no focal fibrosis undergoing catheter ablation, fascicular disease, characterized by fractionated, split, and delayed Purkinje potentials, was found to be present in both patients with a history of cardiac arrest and subsequent identification of ventricular ectopy arising from the Purkinje tissue as clinical ventricular fibrillation triggers and in patients without a history of syncope or cardiac arrest in whom standard ventricular pacing maneuvers induced ventricular fibrillation.^9^ Furthermore, in a study with patients with VT originating from the posterior papillary muscle, the ventricular muscle potential always preceded the Purkinje potentials during PVCs or VT.^18^ Thus, the Purkinje system may also enable a re-entrant mechanism initiated by the papillary muscle, and fascicular PVCs may represent echo beats of the re-entry.

Transient modulators, such as increased sympathetic and/or decreased parasympathetic activity, electrolyte abnormalities, and altered hemodynamics, may also contribute to the arrhythmogenic milieu.

The first case report in this series was a 45-year-old female patient with an out-of-hospital cardiac arrest due to ventricular fibrillation as the first manifestation of bileaflet MVP. Relevant risk markers for MVP-related SCD in this case were female sex, leaflet redundancy (classic MVP, Barlow’s disease), MAD, T-wave inversions in leads III and aVF, and ventricular ectopy. Notably, there was no evidence of focal fibrosis, as indicated by the lack of late gadolinium enhancement on CMR. Assessment of diffuse myocardial fibrosis by T1 mapping imaging was not performed. This case highlights the idea that ventricular fibrillation may occur in the absence of focal fibrosis in patients with bileaflet MVP. Indeed, the presence of bileaflet and redundant mitral valve tissue together with ventricular ectopy and perhaps some transient modulators may constitute an exceedingly dangerous arrhythmogenic milieu on account of several potential lurking mechanisms.

The second case was a 59-year-old male patient with severe MR due to bileaflet MVP with flail posterior leaflet (classic MVP, Barlow’s disease), MAD, pickelhaube sign, and complex ventricular ectopy (polymorphic PVCs and polymorphic NSVT during a treadmill exercise stress test). Moderate-to-severe MR is independently associated with malignant VAs in patients with MVP.^19^ Moreover, MVP-related MR is associated with an increased prevalence of focal fibrosis compared to non–MVP-related MR, localized mostly in the basal inferolateral and basal inferior walls; additionally, focal fibrosis increases with MR severity and is associated with malignant VAs.^20^ Pickelhaube sign, defined as a peak systolic lateral mitral annulus velocity of ≥16 cm/s, with tissue Doppler imaging resembling the “pickelhaube,” a spiked helmet, is a novel echocardiographic marker of arrhythmic risk in patients with myxomatous bileaflet MVP. In fact, patients with pickelhaube sign are more likely to experience malignant VAs and have focal fibrosis on CMR.^21^ Similarly, MAD and its severity are associated with focal fibrosis and VA burden.^22^ Although mitral valve repair should theoretically relieve stretch on the papillary muscles and ameliorate adverse ventricular remodeling, leading to a reduction in VAs, the current literature regarding its role on arrhythmic MVP is controversial. Indeed, mitral valve surgery was associated with a reduction in VAs in a few studies including younger patients (~42 years of age) but not older patients (~62 years of age).^2,23–25^ This is most probably due to the progressive nature of the arrhythmic substrate, as well as the more frequent presence of diffuse fibrosis and idiopathic outflow track ectopy in older patients.^2^ Because of the presence of severe, MVP-related MR, pickelhaube sign, and MAD, it was considered highly likely that the patient had focal fibrosis (substrate). Furthermore, given the presence of complex ventricular ectopy (trigger), the patient was considered as being at very high risk for a fatal arrhythmic event. Therefore, it was decided that the patient should be referred for CMR imaging and emergent mitral valve repair surgery, with the objective of re-evaluating him after these procedures.

## Conclusion

The association of MVP and SCD is supported by several clinical, electrocardiographic, and imaging findings. However, there is no single risk marker, nor any specific combination of them, that has proved to be a consistent predictor of malignant VAs and SCD in patients with MVP. Therefore, further research is needed to validate the existing risk markers, improve our understanding of the underlying arrhythmogenic mechanisms, and establish optimal evidence-based treatment strategies.
